# *FIAT LUX*: The Mullein’s (*Verbascum* sp.) Image and Its Symbology Through History Within the Euro-Mediterranean Culture

**DOI:** 10.3390/plants14213294

**Published:** 2025-10-28

**Authors:** Nicolò Soldovieri, Alessandro Lazzara, Giulia Albani Rocchetti, Flavia Bartoli, Giulia Caneva

**Affiliations:** 1 Department of Science, Roma Tre University, Viale G. Marconi 446, 00146 Rome, Italy; nic.soldovieri@stud.uniroma3.it (N.S.); alessandro.lazzara@uniroma3.it (A.L.); giulia.albanirocchetti@uniroma3.it (G.A.R.); 2 NBFC, National Biodiversity Future Center, Piazza Marina 61, 90133 Palermo, Italy; 3 Institute of Heritage Science (ISPC), National Research Center (CNR), SP35d, 9, 00010 Montelibretti, Italy; flavia.bartoli@cnr.it

**Keywords:** phyto-iconology, plant symbolism, light symbolism, Greek-Roman archaeology, Renaissance, Caravaggio

## Abstract

The plant’s representation had, in the past, a great symbolic relevance, which is now often neglected. The presence and significance of mullein (*Verbascum* sp.) in Euro-Mediterranean art have been investigated, but despite its iconographic importance, a wide analysis of its value and recurrence is lacking. Through a survey of over 5000 artworks, from ancient to modern age, combining digital museum collections and fieldwork, we identified about hundred depictions of *Verbascum*, 64 of which are here reported for the first time. Based on key morphological traits, *V. thapsus* and *V. sinuatum* emerged as the most frequently depicted species, particularly through their basal leaves and inflorescences (especially in modern ages). In archaeological contexts, *Verbascum* overall appears as a symbol of Athena/Minerva, bringers of light, and in funerary settings, such as Apulian vases and tombs, symbolizing new life in the afterlife. After its absence during the Middle Ages, the plant reappeared in the Renaissance, carefully portrayed by notable artists, such as Leonardo, Correggio, Bellini, Dürer, Caravaggio, and Bernini. During this period, mullein is often associated with Christ and St. John the Baptist, reinforcing its symbolism of light and spiritual elevation. Other representations also occurred in the subsequent centuries, but in a renovated vision of the natural world.

## 1. Introduction

Plants have always held a profound place in the history of human existence: they nourish us, provide us with materials, and, in their quiet beauty, carry layers of symbolic meaning. This dual role, both practical and spiritual, has made them a frequent muse in art [1,2,3]. Whether serving as decoration, symbolism, or vivid description, plants assume diverse roles that vary according to the purpose of the artwork, its typology, and the cultural context in which it was made.

In ancient cultures such as those of the Egyptians, Persians, Romans, and Greeks, the selection and depiction of plant motifs in architecture and art were far from arbitrary [4,5,6,7,8,9]. In these traditions, nature was not merely observed but deeply felt; it represented an expression of a universe where the divine governed all living things, and every leaf and blossom spoke of deeper truths. Natural phenomena were seen as manifestations of divine will, with tangible consequences for human life, whether as blessings or punishments [2,10,11,12,13,14]. Despite this, floral elements in art, in both paintings or archaeological artefacts, are commonly misinterpreted or regarded as purely decorative. Such modern interpretations fail to acknowledge the profound cultural and spiritual significance that plants once held [5].

In the Euro-Mediterranean regions, especially with the rise of Christianity, the once intimate connections between humanity and nature weakened due to a growing separation between the divine and the natural world [15]. During the Middle Ages (6th to 15th century CE), sacred figures such as Christ, the Virgin Mary, and the Saints became central subjects in art. In contrast, natural representations were relegated to the margins, losing both their accuracy and symbolic prominence.

When considering the interpretation of such elements, it is worth noting that although botanical representations have frequently appeared in art throughout history, their identification, which is essential for understanding this visual and symbolic language, is not always straightforward. An incorrect identification of these elements can give rise to a misunderstanding of their original meaning.

**Figure 1:**
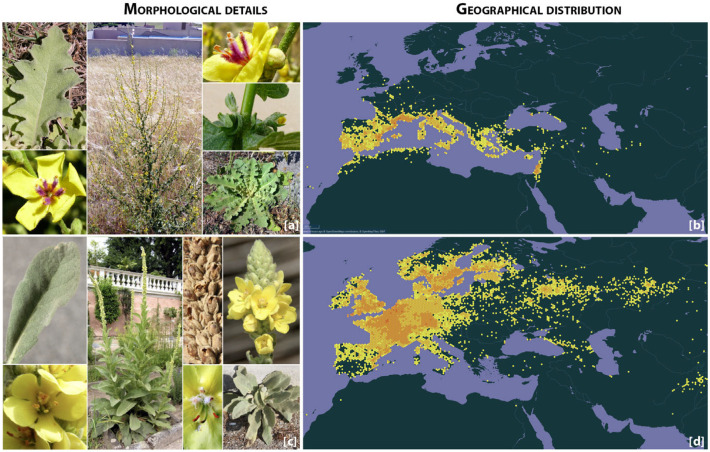
Morphological details (Copyright: Dip. Scienze della Vita, Università degli Studi di Trieste; Author: Andrea Moro; CC-BY-SA 4.0) of *Verbascum sinuatum* (**a**,**b**) and *V. thapsus* (**c**,**d**). Present geographical distributions (https://doi.org/10.15468/ab3s5x) of *V. sinuatum* (**a**,**b**) and *V. thapsus* (**c**,**d**).

Within the panorama of studies on plant iconography in art, the representation of mullein (genus *Verbascum*) appears largely neglected, both in antiquity [16,17] and in the modern age [18]. Yet, the genus *Verbascum* seems particularly noteworthy in the Euro-Mediterranean context, where its widespread popular uses suggest that its artistic representation should be of some relevance. The genus comprises a wide group of species, at least 450, within the Scrophulariaceae family, with the highest species diversity found in the Mediterranean region [19] (Figure 1). These plants, with a typical columnar or ramified yellow inflorescence, thrive not only in natural habitats but also in anthropogenic environments, showing remarkable ecological adaptability. Pliny the Elder (NH, 25.73) described only two species, typically identified as *V. thapsus* and *V. sinuatum*, whose general characteristics and current distributions are shown in Figure 1. These plants had ethnobotanical and traditional uses linked to their name etymology. The term “Verbascum” is thought to derive from a variation of *barbascum*, which originates from the Latin term “*barba*” (“beard”), likely referring to the plant’s fuzzy appearance. The common name “mullein”, on the other hand, comes from the Middle English “*moleyne*” and Old French “*moleine*”, both derived from the Latin “*mollis*” (“soft”), referred to the soft texture of the plant’s leaves [20]. More significantly, these plants were associated with fire and light, as suggested by the ancient Greek name “*phlomos*” (*φλόμος*), related to “*phlox*” (*φλόξ*, “flame”), and the Latin epithet “*candela regia*”. In ancient Rome, *Verbascum* stems were used as torches during funerals and ceremonial rituals [21].

We therefore argue that the assumption of a minor artistic role for this plant is likely unfounded, as its presence was often misidentified or neglected due to a modern detachment from natural imagery. For example, the basal leaves on capitals of the Temple dedicated to Minerva in the archaeological park of San Leucio (4th century BCE, Canosa of Apulia, Italy) are usually described as related to *Acanthus*, rather than those of mullein [22]. A similar misattribution to acanthus leaves occurs in the plants carved on the fronton ornamentation of the *Castrum Minervae* sanctuary (4th century BCE, Castro, Apulia, Italy) [23]. Likewise, on Apulian vases from around 440 BCE, elements often labelled as “acanthus calyx” may in fact represent *Verbascum* as generative elements [24]. Only recently, *Verbascum sinuatum* has been proposed as the actual subject of these depictions, likely due to its columnar yellow inflorescence and its use as a torch, evoking the symbolic role of “bringing light” [25]. This interpretation provides a distinct, even if in a certain sense comparable, meaning to that of the *Acanthus* motif, traditionally associated with the idea of “rebirth” [26,27].

In modern art, scholars have increasingly noted the presence of *Verbascum*, especially in sacred imagery, from the paintings of Caravaggio [28,29,30,31], to depictions among the Caravaggeschi, such as Giuseppe Vermiglio [32], and in the famous fountain of the Four Rivers of Gian Lorenzo Bernini [7]. However, while there is evidence that *Verbascum* has been portrayed in art, a more exhaustive analysis of its recurrence is still lacking. As previously cited, most scholars have emphasized that this plant was not merely decorative but carefully chosen and symbolically significant. It is therefore worthwhile to explore the origins of its visual representation and to evaluate its frequency and meaning across historical periods. This study aims to analyze: (i) the presence and recurrence of mullein in artistic and historical representations within the Euro-Mediterranean culture; (ii) the figurative context of these representations, to interpret the reasons for its selection over other species, according to possible variations in terms of symbolism and perception of nature throughout the considered chronological periods.

Through a comprehensive survey of plant imagery in artworks, from ancient to modern age, this study highlights that mullein appears more frequently in art than previously recognized and underscores the enduring recurrence of its symbolic meaning through the centuries.

## 2. Results

### 2.1. Plant Identification and Its Recurrence in Artworks

#### 2.1.1. Identification

**Figure 2:**
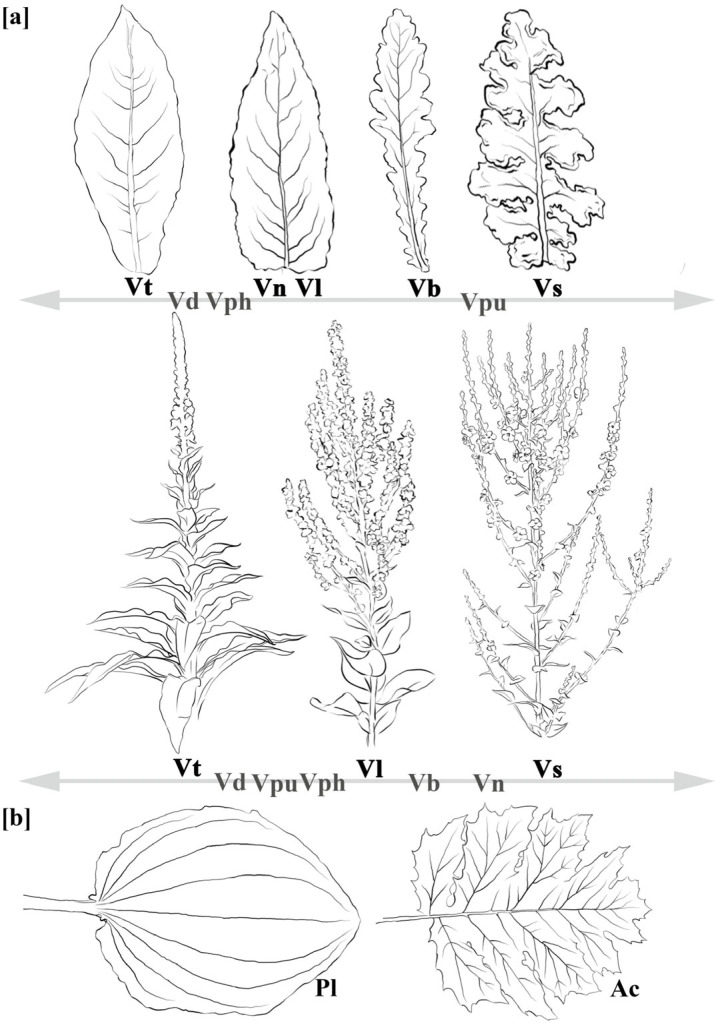
Morphological features of leaves and inflorescence of (**a**) the eight most common *Verbascum* species (acronyms: Vt: *V. thapsus*; Vn: *V. nigrum*; Vl: *V. lychnitis*; Vs: *V. sinuatum*; Vd: *V. densiflorum*; Vpu: *V. pulverulentum*; Vph: *V. phlomoides*; Vb: *V. blattaria*); (**b**) leaves of *Plantago* (Pl) and of *Acanthus* (Ac) genera.

To accurately identify the *Verbascum* species represented in artworks, we extracted the most widely distributed species in Europe from the GBIF dataset (https://doi.org/10.15468/4qdfhd; accessed on 31 March 2025), retrieving a total of 549,681 occurrence records. From these, we selected species with more than 10,000 documented records, resulting in the following list: *Verbascum thapsus* L. (154,324 records), *V. nigrum* L. (134,522), *V. lychnitis* L. (62,018), *V. sinuatum* L. (36,047), *V. densiflorum* Bertol. (30,325), *V. pulverulentum* Vill. (24,698), *V. blattaria* L. (21,291), and *V. phlomoides* L. (17,122). Species identification in the artworks was based on an analysis of key morphological traits, as outlined in Supplementary Table S1. Among the collected artworks, the most identifiable elements corresponded to two widely distributed *Verbascum* species in the Euro-Mediterranean area: *V. thapsus* L. and *V. sinuatum* L. For the uncertain elements, we considered a *thapsus*-like or *sinuatum*-like model, when shared morphological characteristics close to these two species. Both species are native to the Mediterranean region and display common traits, including an erect inflorescence stem bearing densely clustered yellow flowers (Figure 2).

The primary observable distinction between these two species lies in leaf morphology (inflorescences also vary, but they are not always depicted). Both species produce a basal rosette of leaves covered in dense trichomes, giving the leaves a silvery appearance, and have cauline leaves extending up the stem. However, the basal leaves of *V. sinuatum* are oblanceolate and feature irregular, triangular lobes that are distinctly undulated, crenate, or occasionally edentate. In contrast, *V. thapsus* typically displays larger and longer basal leaves, with upper stem leaves ranging from oblong to oblanceolate, and having simpler, more uniform margins, features that aid in their identification (Figure 2a). Leaves’ characteristics, with their possible variations, were essential for distinguishing *Verbascum*’s leaves from those of other similar or commonly depicted species. Notably, misidentifications have frequently involved *Acanthus mollis* L. in the ancient period, and *Plantago major* L. in modern representations (Figure 2b). These species share certain traits with *Verbascum*, such as forming basal rosettes and producing flowers on an erect spike, yet they differ in other characteristics. *Acanthus*’ leaves are significantly larger, more deeply lobed or toothed, and the flowers, when visible, vary in both structure and color. Similarly, *Plantago major*, a perennial herb native to Eurasia, also forms a basal rosette, but its leaves are broader, ovate to elliptic (or occasionally cordate), and notably lack the dense pubescence characteristic of *Verbascum* species.

#### 2.1.2. Recurrence

**Figure 3:**
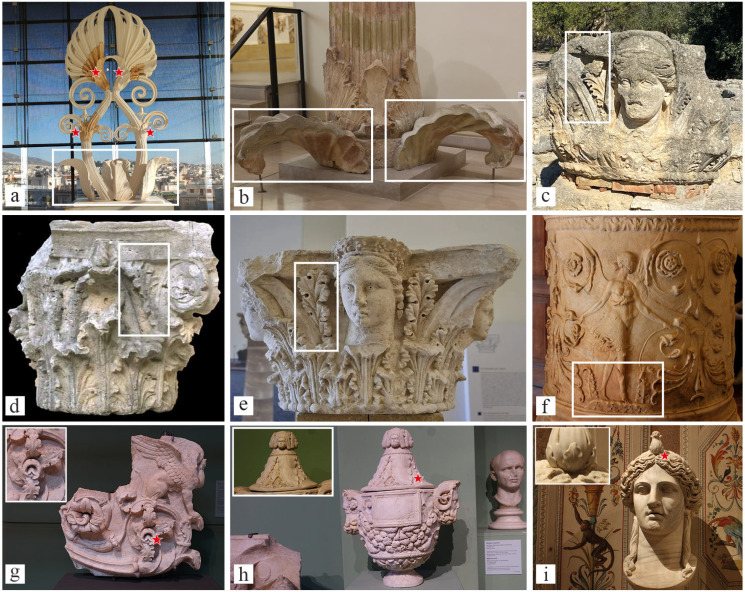
Representative examples of the Greek and Roman Classical Period depicting *Verbascum* species. (**a**) Akroterion of Parthenon—Museum of the Acropolis (Athens); (**b**) The column of the dancers—Archaeological Museum of Delphi; (**c**) Capital with female figure—Archaeological park of San Leucio (Canosa di Puglia); (**d**) Corinthian capital—Palestrina (Rome); (**e**) Figurate capital—Archaeological museum “F. Ribezzo” (Brindisi); (**f**) Cylindrical base with dancers—Museo Nazionale Romano, Palazzo Altemps (Rome); (**g**) Frieze with acanthus volutes—Centrale Montemartini Museum (Rome); (**h**) Cinerary vase—Centrale Montemartini Museum (Rome); (**i**) Colossal Head of a Divinity—Galleria Borghese (Rome). Red stars and white squares mark the precise presence of the *Verbascum* specimen depicted. Images by the authors or from copyright-free web sources.

**Figure 4:**
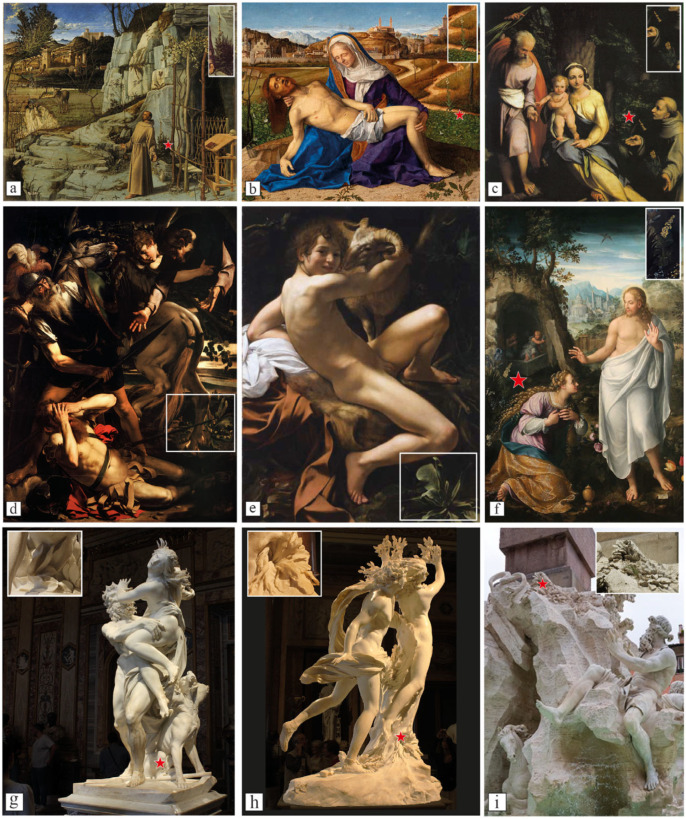
Representative examples of Renaissance and modern era artworks depicting *Verbascum* species. (**a**) Saint Francis in the Desert by Giovanni Bellini; (**b**) Pietà by Giovanni Bellini; (**c**) Rest on the Flight into Egypt with St. Francis by Correggio; (**d**) The Conversion of Saint Paul by Caravaggio; (**e**) San Giovanni Battista by Caravaggio; (**f**) Noli me tangere by Fede Galizia; (**g**) Rape of Proserpine by Gian Lorenzo Bernini; (**h**) Apollo and Daphne by Gian Lorenzo Bernini; (**i**) Fountain of four rivers by Gian Lorenzo Bernini. Red stars and white squares mark the precise presence of the *Verbascum* specimen depicted. Images by the authors or from copyright-free web sources.

**Figure 5:**
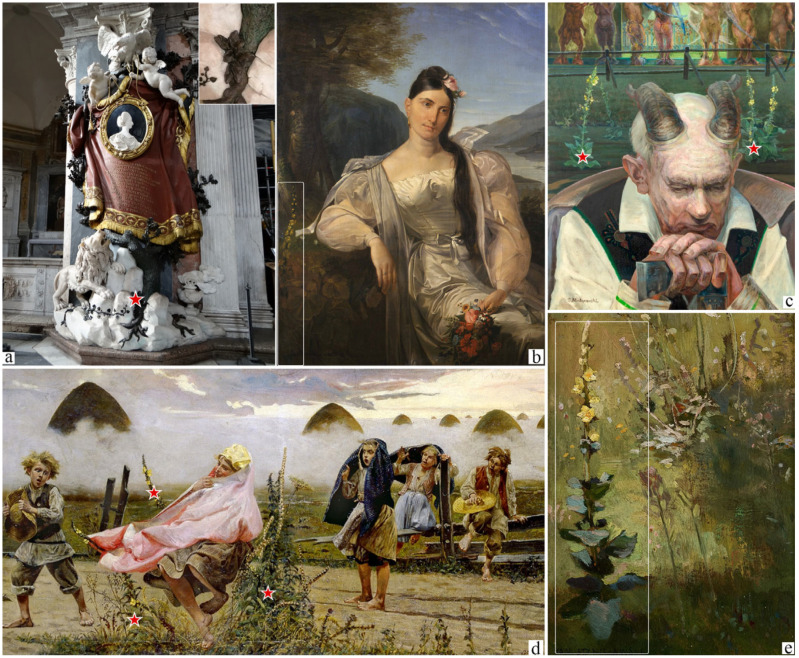
Representative examples of eighteen- and nineteen-century artworks depicting *Verbascum* species. (**a**) The tomb of Maria Flaminia Odescalchi Chigi by Paolo Posi; (**b**) Portrait of the singer Giuditta Pasta by Giovanni Molteni; (**c**) Portrait of a Faun by Jacek Malczewski; (**d**) The Goddess in the Mullein by Jacek Malczewski; (**e**) Mullein by Jan Stanislawski. Red stars and white squares mark the precise presence of the *Verbascum* specimen depicted. Images by the authors or from copyright-free web sources.

Upon reviewing the previously cited resources, comprising approximately 5000 artworks featuring plant elements, and despite the inherent challenges in species identification, a careful morphological analysis allowed us to identify mullein across a relatively wide range of artistic media and historical contexts (Table S1; Figure 2). While *Verbascum* was present in a relatively limited number of artworks, it consistently appeared in specific contexts, resulting in 97 archaeological elements or artworks from various museums and sites (Figure 3, Figure 4 and Figure 5), 70% of which are new records.

Among these 97 mullein occurrences, 33 belonged to the archaeological materials from Greek, Roman and ancient cultures of southern Italy. Within this subset, 27 recurrences (82%) are described here for the first time (Table 1, Figure 3, Supplementary Table S2). For the Renaissance and Modern periods, we identified 56 artworks containing *Verbascum*, of which 31 (55.4%) represented previously undocumented occurrences. In contrast, no depictions of mullein were found in artworks from the Middle Ages (Table 2, Figure 4, Supplementary Table S3). For the eighteenth and nineteenth centuries, the number of occurrences diminishes, and we selected eight main artworks featuring *Verbascum*, with 3 (37.55%) newly reported in this study (Table 3 and Table S4, Figure 5).

Overall, the species were not equally represented in the dataset: *V. thapsus* appeared in 55 instances, while *V. sinuatum* was identified in 36, with only one artwork in which both of them are depicted. Two artworks depicted other *Verbascum* species (*V. densiflorum*, *V. nigrum*), and three remained undetermined at the species level.

**Figure 6:**
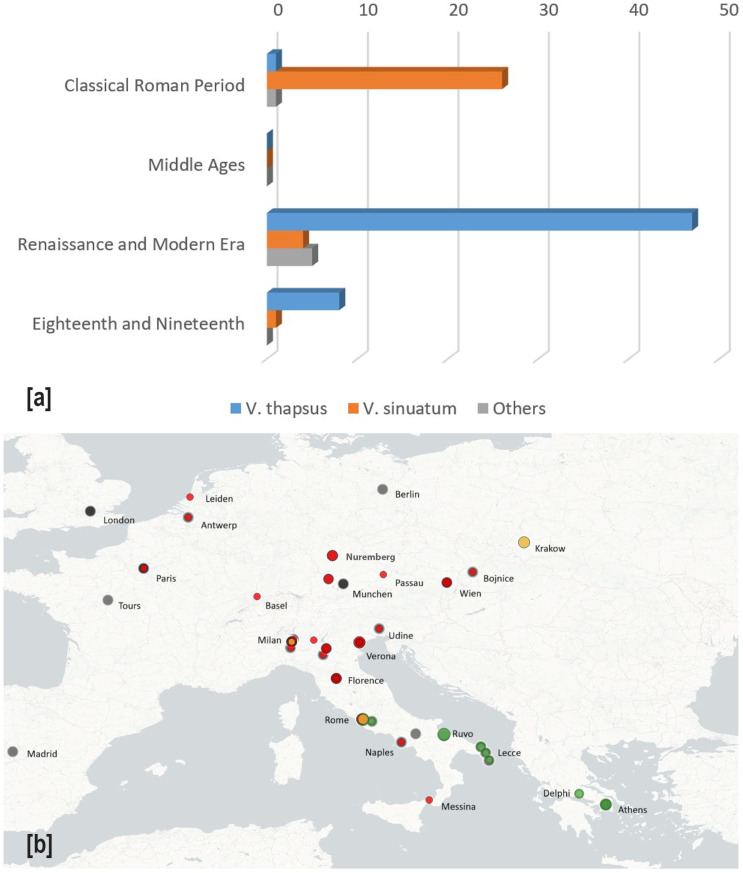
(**a**) Chronological occurrence of *Verbascum* species through the different analyzed periods and (**b**) provenance (green—Classical Greek and Roman Period, red—Renaissance and Modern era, yellow—eighteenth and nineteenth centuries) and present location (black) of the analyzed artworks. The width of the dots is proportional to the number of occurrences.

The analysis of chronological trends revealed two distinct patterns in the representation of the two *Verbascum* species. *V. sinuatum* was the main species depicted during the Archaic period, but its frequency declined in subsequent eras. In contrast, *V. thapsus* was rare in the earliest artworks yet became increasingly prominent over time, particularly during the Renaissance, when its representation significantly surpassed that of *V. sinuatum* (Figure 6a).

The analysis of geographical distribution revealed two distinct patterns in the representation of the analyzed artworks. The works of art of the Classical Greek and Roman Period are strictly related to the Mediterranean area, whereas those of the Renaissance and Modern Era cover most of the Euro-Mediterranean area (Figure 6b).

### 2.2. Mullein’s Association and Interpretation of Symbology

When comparing ancient and modern periods, we observed a notable difference in the subject of artworks depicting *Verbascum* species. However, a common significance can be found.

**Figure 7:**
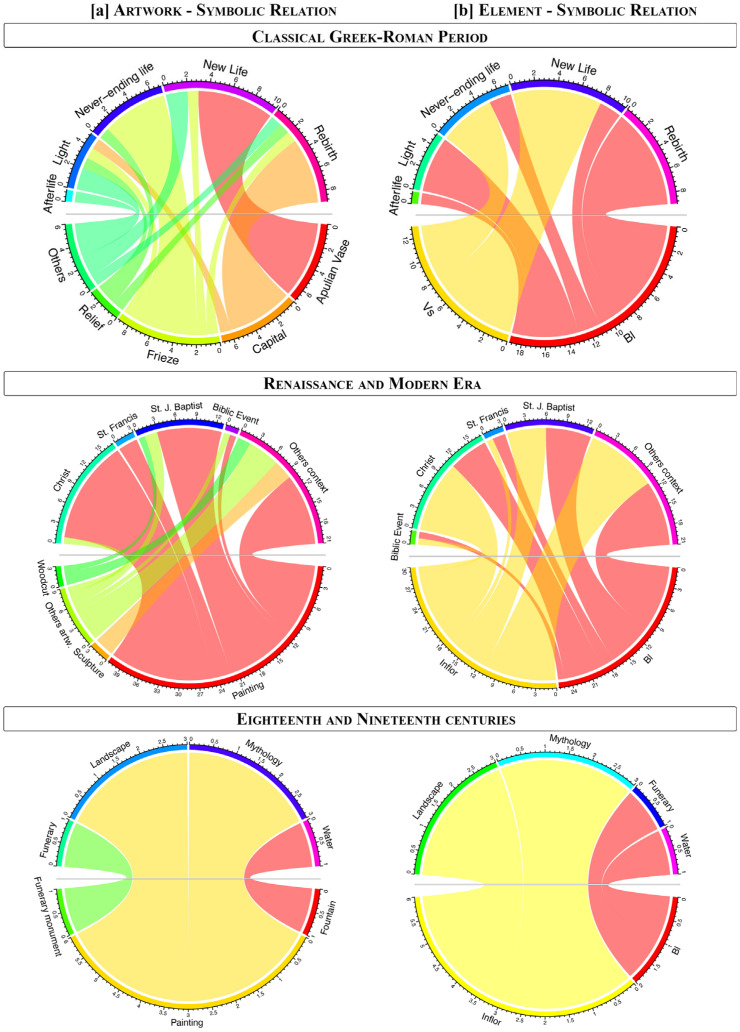
Comparison between (**a**) the typology of artwork and the symbolic relation, and (**b**) the element depicted and the symbolic relation in different periods analyzed.

In ancient times, mullein mostly appeared represented in funerary contexts, such as on Apulian painted vases, although its association with the goddess Minerva and her symbolic significance should not be overlooked. In contrast, the modern period exhibits a broader variety of artwork typologies featuring *Verbascum* (Figure 7a).

During the Renaissance and modern age, most representations of *Verbascum* appear in Christian-themed paintings. Mullein is often associated with Christ (especially in the scenes of Nativity, Rest on the Flight into Egypt, Crucifixion, and Compassion; 18 out of 56 artworks) and St. John the Baptist (12 out of 56 artworks), or certain Saints, such as St. Paul, St. Francis and St. George (Figure 7). Finally, during the last period, encompassing the eighteenth and nineteenth centuries, *Verbascum* images appear to have lost their previous relevance. Its presence, again limited to a few examples in important artworks, still appears in contexts that are partly continuations of earlier traditions (e.g., funerary settings), and partly in new contexts not previously associated with the plant (e.g., part of the landscapes and fountains). Indeed, its magic power is still detectable, with a renewed attention to mythology by a group of Polish painters, such as Jacek Malczewski and Jan Stanislawski.

A notable difference was observed in the depiction of *Verbascum* morphological parts across historical periods and contexts. In ancient artworks, the basal leaves and vegetative system, which predominate, are associated with metamorphic or funerary contexts (Figure 7). In contrast, in the modern period, depictions are dominated by the basal leaves, followed by the inflorescence, both of which are strongly linked to Christian figures, particularly Christ, St. John the Baptist, and St. Francis (Figure 7). The representation of *Verbascum* varies in early modern depictions of Christ, where different plant parts appear in distinct iconographic contexts. A detailed analysis of these artworks, particularly those illustrating events from the life of Christ, revealed a discernible pattern. Approximately 80% of the depictions of the basal rosette are associated with scenes involving the Nativity or the youth of Christ, such as the biblical event of the Rest during the Flight into Egypt. The inflorescence, on the other hand, appears in about 67% of scenes depicting the later part of Christ’s life, including the Flagellation, Crucifixion, Entombment, Compassion, and Resurrection. The basal leaves, consistently present across both ancient and modern periods, are the most frequently represented plant structure overall. The inflorescence, while rare in ancient depictions, becomes more prominent in modern artworks, where its occurrence nearly equals that of the basal rosette. Notably, depictions of only cauline leaves are absent in modern representations. It is only in the last period (eighteenth and nineteenth centuries) that this tendency shifts, with the inflorescence becoming the most frequently depicted part of the plant, appearing in 6 out of 8 artworks (Figure 7).

## 3. Discussion

Combining the review of the literature with the morphological and iconological analysis, an extensive dataset that spans both ancient and modern artistic representations was established and analyzed, highlighting new recurrences, patterns of association, shifts in contextual use, and changes in the symbolic meaning of this species that would have otherwise remained unnoticed.

### 3.1. Identification

Challenges in the identification arise from factors such as the accuracy of representation, the expressive means and the artistic technique adopted, the purposes of the artwork, and the historical period in which it was made. On the other hand, the identification of natural elements can be facilitated by distinct plants and animals’ morphologies, the realism of their depiction, and the use of color and three-dimensionality, which enhance their visual recognition [16]. Then, the final wide number of 97 occurrences cannot be considered a definitive data on mullein presence in the Euro-Mediterranean artworks, since we did not check the entire artistic production, but only a significant part. Indeed, our aim was initially to check its presence across various ages, using a consistent and representative amount of data, and later to analyze its iconographic significance. The high number of new occurrences (68), approximately double concerning those previously recognized, can be attributed in part to earlier misinterpretations in iconographic analysis, including incorrect attributions to *Plantago* [18] and *Acanthus* [23], as well as the omission of certain details, as in the case of Botticelli’s Spring [44]. However, other significant cultural factors, such as rationalism and illuminism, contributed to a decline in both the use of natural elements and their visibility in modern visual culture. When species-level identification was possible, *V. sinuatum* was more frequently depicted in ancient times compared to *V. thapsus*, likely reflecting its broader occurrence in arid and Mediterranean areas of Southern Italy and Greece (see Figure 1, where many of the artworks dated to this period have been carried out; see Figure 6b).

### 3.2. Symbolic Aims and Recurrences Throughout History

The recurrence of *Verbascum* is closely linked to its symbolic value, which evolved throughout history. Beyond Caravaggio, Giovanni Bellini, Gian Lorenzo Bernini, and other previously cited artists [7,27,30,31,32], the consistent depiction of *Verbascum* also by other artists suggests that it was not merely an ornamental feature, but the plant was intentionally selected for its symbolic resonance. Its consistent appearance across diverse geographical regions and chronological periods challenges the prevailing view of plants in art as decorative elements alone. Instead, mullein appears to hold deep cultural and spiritual significance.

The analysis of the specific plant parts depicted, the typologies of the artworks, and the contexts in which *Verbascum* appears reveals a consistent symbolic thread that stretches from antiquity through the modern period. As previously proposed [25], its association with themes of light and protection likely derives from multiple interrelated factors. The yellow color of its flowers evokes the sun, and its ecological preference for open, sunlit environments aligns with its symbolic identity as a plant of illumination. Moreover, its ability to sprout from long-life seeds may have contributed to associations with rebirth and spiritual resilience [45].

Its use as a torch of flame-bearer is documented in ancient texts and supported by the analysis of Austrian and South German panel paintings. In these traditions, common names such as *kerze*, *kertzenkraut*, and *himmelprant* link the plant with candles and divine light, reinforcing its association with religious and sacred imagery [34].

Additionally, *Verbascum* has a long-standing medicinal history. References to its healing properties can be found in classical texts such as Pliny the Elder’s *Naturalis Historia* and in medieval sources like Hildegard von Bingen’s “*Physica*” under “De Wullena”, which probably referred to *V. thapsus* L. [46]. In artworks such as Florence’s Fontana del Porcellino, *Verbascum* appears alongside other wild plants linked to Tuscan folk medicine [16]. Within the Scrophulariaceae family, many plants have been evaluated for their medical properties and have long been used in both folk and conventional medicine [47,48]. Of these, the most significant species historically used for medical purposes is *Verbascum thapsus* [21], but also *V. sinuatum*, particularly for treating respiratory issues. The leaves and flowers are known for their expectorant and demulcent effects, which help alleviate conditions like bronchitis, dry coughs, asthma, and hoarseness. The plant’s soothing action on the respiratory system is attributed to its mucilaginous content, while saponins are believed to contribute to its expectorant properties [49]. Mullein’s medicinal uses extend beyond the respiratory system as it is known to possess anti-inflammatory, analgesic, antiseptic, and antispasmodic properties, as well as calming and sedative effects. Though the plant offers a broad spectrum of therapeutic benefits, caution is advised with the seeds, which are toxic and should not be used in preparations [21,50].

### 3.3. Ancient Art

In ancient art, *Verbascum*, though often misinterpreted as *Acanthus* calyx, was first identified in the *Akroterion* of Parthenon (5th Century BCE) (Figure 3a). It is also frequently represented in Apulian vase paintings, serving as a decorative motif surrounding disembodied female heads. In Attic vase painting, similar female heads appear in *anodos* scenes, which depict deities emerging from the underworld [51]. Among the thousands of extant vases featuring such motifs, nearly all with confirmed provenance have been recovered from funerary contexts. Their sepulchral function is further supported by morphological features, such as intentional perforations that made them unusable for daily life [52]. The surrounding flora in these compositions suggests a symbolic interplay between death and regeneration. Positioned directly above grave monuments, these figures that emerge from flowers serve as strong metaphors for rebirth and the promise of an afterlife [52]. Within this framework, the symbolic value of *Verbascum* may be further understood through its ancient Greek designation and its association with the *caduceus* carried by Hermes [53,54]. Hermes, the oracular deity who presided over the passage between the realms of the living and the dead, used his rod to guide departed souls to the underworld [55]. The association of *Verbascum* with Hermes and his necromantic functions, therefore, reinforces the plant’s funerary symbolism and emphasizes its functions as a mediator between life and death.

This symbolic framework remains visible in later artistic traditions, where iconographic references to mythological themes, particularly involving Demeter and Persephone, perpetuate ancient funerary motifs. While the association between *Verbascum* and these divine female figures has not been widely explored in prior scholarship (aside from isolated references, e.g., [25]), it presents a promising direction for future research. The frequent appearances of female heads on Apulian vases, long interpreted as divine figures, including Persephone, suggest a strong thematic alignment. Given her role in the mythological cycles of death and seasonal rebirth, Persephone’s cyclical narrative may align closely with *Verbascum’s* symbolic connotations of regeneration and light [51].

This connection extends into Dionysian contexts as well. Dionysus, a deity frequently featured in funerary iconography such as the volute krater attributed to the Darius Painter depicting him reaching toward Hades and Persephone, was venerated in Italic cultures as a guarantor of a blessed afterlife [56]. In this symbolic framework, *Verbascum* may serve as a visual mediator, embodying the themes of life, death, and cyclical renewal. In ancient funerary artworks, *Verbascum* is typically shown in its vegetative form, most often as basal leaves, while the inflorescence, strongly associated with light and spiritual revelation, is conspicuously absent. This omission may not be incidental but instead a deliberate iconographic strategy. The unflowered state of the plant may symbolize the latent hope for renewal, reinforcing the funerary theme of rebirth and the aspiration to “see the light” once more in an afterlife. Indeed, beyond Demeter and Persephone, *Verbascum* also appears to be associated with Athena (or her Roman counterpart, Minerva) and with Apollo. The linkage with Athena is well evident in the magnificent *Akroterion* of Parthenon, in the Acropolis of Athens, but also in the *Athenanion* frieze of the *Castrum Minervae* sanctuary and the temple dedicated to Minerva in the archaeological park of San Leucio (Figure 3c) [25].

In Homeric literature, Athena is strongly linked to light. In The Odyssey (book 19), she is described as bearing a “golden lamp” (*chryseon lychnon*) that emits “very beautiful light” [57]. Her most enduring epithet in Greek poetry, *glaukopis* (*γλαυκώπις*), often translated as “bright-eyed” or “flashing-eyed”, derives from *glaukos* (*γλαύκος*, “shining” or “silvery”) and ops (*ώψ*, “eye” or occasionally “face”). Athena’s symbolic scope includes wisdom, natural fertility, and protective power, attributes shared by Demeter and the Great Mother [58]. This multifaceted role is further reflected in the Roman conception of Minerva, who similarly embodied knowledge, intellectual clarity, and strategic insight. Her association with light, whether literal, as in the golden lamp, or metaphorical, as in “illumination” of the mind, resonates with the visual and symbolic presence of Verbascum in sacred and intellectual contexts, such as temple decoration and iconography related to divine wisdom. Apollo, as the god of the sun, had an evident connection with the light, similar to that of victory, in both Greek and Roman cultures (for example in Pythian Games, where winners were awarded a wreath of laurel, and in Rome, where emperors wore laurels crowns as symbols of triumph) [59].

### 3.4. Middle Age

Notably, *Verbascum* is nearly absent, or not clearly detectable, in medieval artistic representations, a fact that may reflect both stylistic and symbolic factors. Early medieval landscapes were highly schematic, often prioritizing allegory over naturalism. These works tend to appear rigid and stylized, lacking a realistic perspective and anatomical accuracy. As Clark and Taylor (1976) [60] noted, only at the end of the Middle Ages, artists began to adopt perspective and greater anatomical accuracy, facilitating a more realistic depiction of flora. Moreover, during the medieval period, the allegorical interpretation dominated artistic expression, leading to what White (1947) [61] termed “shadow-art,” in which distorted natural forms were used not for their mimetic accuracy but to convey theological or supernatural meanings. Yet even in the absence of visual depictions, *Verbascum* retained symbolic resonance in medieval literature. It was widely believed to possess protective powers: a medieval herbal, for instance, records: “if a man carries a twig of this plant (mullein), he will not be frightened by any terror, nor will a wild beast harm him, nor will any evil come near” [62]. This belief in its spiritual efficacy persisted across time, evolving in tandem with its representation in art.

### 3.5. Renaissance and Modern Era

This symbolic thread extends into the modern period, where *Verbascum* begins to appear in association with Christian figures, particularly Christ and St. John the Baptist. Although the religious framework has shifted, the plant retains its core symbolic associations, especially those linked to light, renewal, and salvation. In both ancient and Christian iconographies, *Verbascum* functions as a visual metaphor for transcendence, intercession, and spiritual illumination. In the Gospel of John (8:12), Christ proclaims: “I am the light of the world. Whoever follows me will never walk in darkness but will have the light of life.” Similarly, in John 5:33–35, the Baptist is described in explicitly luminous terms: “He was a burning and shining lamp, and for a while you chose to enjoy his light”. This theological emphasis on light is echoed in the visual use of *Verbascum*, particularly in how different plant parts are associated with different phases of Christ’s life. In Nativity and childhood scenes, the plant is most often shown in its vegetative state, with a focus on the basal rosette emphasizing the latent promise of salvation.

By contrast, episodes associated with the Passion, Crucifixion, Entombment, and Resurrection (Figure 4b,f), the culmination of Christ’s salvific mission, are more frequently linked to the flowering inflorescence. This progression reflects theological narratives in which divine light, initially veiled, is fully revealed through sacrifice and resurrection. As Rust (2011) [63] notes, “gospel light” functions as both guidance and revelation, and the symbolic flowering of *Verbascum* in these scenes reflects that spiritual climax [63]. The mature inflorescence becomes a visual metaphor for spiritual fulfilment and the manifestation of divine light. This symbolic development across time underscores *Verbascum’s* persistent role as a botanical signifier of growth, sacrifice, and transcendence, even as its interpretive clarity becomes increasingly veiled by changing artistic conventions.

The association between mullein and St. John the Baptist was previously explored by White (1996) [36] through an analysis of several works by Albrecht Altdorfer. White notes that, like the Baptist, mullein was traditionally regarded as both a healer and a protector. It was often gathered on the Feast of the Nativity of St. John the Baptist and passed through bonfires to ward off evil spirits. This ritual usage further cements the association between the plant and the saint’s role as a spiritual guardian. In Altdorfer’s Beheading of Saint John the Baptist, a *Verbascum* without its flowering stalk lies beneath the saint’s severed head, symbolically mirroring his martyrdom. Yet nearby, a second specimen blooms, affirming the enduring power of the *lucerna mundi*, the “lamp of the world” [40]. The same symbolic meaning can also be observed in St. Paul’s Conversion and in the Ecstasy of St. Francis, where the idea of the light of faith is conveyed through the deliberate selection of this species at the bottom of the scene (Figure 4d).

This role of the plant as a bearer of light is visually emphasized in works like Gian Lorenzo Bernini’s Fountain of the Four Rivers, where we already found that the plant appears opposite a serpent, a traditional symbol of evil. In this context, *Verbascum* represents divine protection associated with the Christian Church, reflecting longstanding associations of the plant with illumination and protection [7]. In a broader assessment of Gian Lorenzo Bernini’s works, this significance also emerges in the Rape of Proserpina and in the laurel metamorphosis depicted in the famous group of Apollo and Daphne. Here, mullein is prominently represented among the rocks at the base of these mythological subjects, symbolizing the boundary between the Underworld and the Earth, and delineating the threshold between Light and Darkness (Figure 4g,h).

### 3.6. Eighteenth and Nineteenth

During such centuries, representations of mullein can be detected with lower frequency, appearing in contexts that reinforce its traditional associations, such as funerary scenes and symbols of protection. However, many paintings from this time also begin to depict *Verbascum* as an element of the landscape, gradually losing its deeper symbolic value. This shift reflects a broader change in how plant imagery is understood in our contemporary culture, a change that complicates our ability to decode the hidden language of symbolism. Interestingly, the late nineteenth century also saw the emergence of renewed interest in plant symbolism, coinciding with the rise of Symbolism as an artistic movement. Originating in France in 1886, Symbolism rejected materialism, objectivity and rationalism, favoring instead the spiritual, the emotional, and the metaphysical sphere. This movement extended its influence beyond France, notably reaching Poland, where it found the greater expression in the works of artists like Jacek Malczewski [62].

## 4. Materials and Methods

### 4.1. Data Collection

The first step involved a comprehensive bibliographic review using Google Scholar, Scopus, and Web of Science (WOS) to identify existing studies and references documenting the presence of *Verbascum* species in artworks. A preliminary collection of artworks from various Euro-Mediterranean cultures was then carried out, focusing on four main historical periods: the ancient Greeks and the Classical Roman Period (encompassing the Archaic era through the Roman Imperial Age), the Middle Ages (including Early Christian, Byzantine, Romanesque, and Gothic art), the Renaissance and the Modern era (including Baroque and Post-Baroque periods), until the eighteenth and the nineteenth centuries. For each period, the representation of mullein in art was examined in relation to the representation context and the typology of the artworks.

For the Classical Period, we initially re-examined the database developed by Kumbaric and Caneva (2014) [17], which focuses on plant iconography in Roman sculptures, to assess the possible presence of mullein. This database includes works preserved in major Museums and Archaeological Parks in Rome: Vatican Museums, Capitoline Museums, the Imperial and Roman Fora, the National Roman Museum, the Archaeological Parks of Ostia, and the Appia Antica, as well as monuments like the Temple of Hercules Victor (Ercole Vincitore) and the fragments of the Temple of Apollo Sosiano.

Additional sources included the literature on Pompeii and Herculaneum [64,65], such as those of the Archaeological Museum of Naples (MANN) [2], and recent archaeological findings related to the Italic Pre-Roman populations of Southern Italy [25]. In this regard, we consulted the general catalogues of the Italian Ministry of Cultural Heritage (Catalogo Generale dei Beni Culturali, https://catalogo.beniculturali.it), filtering results by Italian region, chronological period, and cultural context.

Between September and December 2024, we conducted on-site visits to the aforementioned Italian museums referenced in the initial databases, searching for additional instances of mullein representation. During these visits, we collected photographic documentation and gathered available chronological and contextual information. Other relevant museums, such as the Vatican Museums and Borghese Gallery (Rome, Italy), were also included in the survey.

For the ancient Greek culture, during May and July 2025, we also considered the main archaeological areas and museums of Athens (Museums of ancient Agorà of Athens, Museum of the Acropolis, National Archaeological Museum of Athens, Museum of Cycladic art and the Archaeological area and Museum of Delphi).

For later periods, the dataset was expanded to include sculptures and paintings. Additionally, online collections from major Italian institutions, such as those of the Pinacoteca di Brera (Milan, Italy) and the Uffizi Gallery (Florence, Italy), were explored (https://pinacotecabrera.org; https://www.uffizi.it).

For each artwork, provenance has been specified. When explicitly stated in the artwork details, provenance refers to the location where the artwork was created. In cases where this information was unavailable, provenance was interpreted as the cultural context in which the artist worked or received training, as this may have influenced the creation of the artwork. Additionally, the current location of each artwork was noted. To evaluate whether the depiction of *Verbascum* was limited to certain geographical areas or more widely shared across European artistic traditions, we extended our analysis to include works by non-Italian artists, particularly Flemish, German, and French painters.

This phase included the online collections of the Alte Pinakothek (Munich, Germany), Kunsthistorisches Museum (Wien, Austria), Prado Museum (Madrid, Spain), Louvre Museum (Paris, France), and the National Gallery (London, UK).

Filtering tools were applied to focus on works corresponding to the relevant time periods. In cases where *Verbascum* was identified, the broader corpus of the artist’s work was examined to determine whether its appearance was isolated or part of a recurring motif. All artworks identified during the bibliographic review and fieldwork were systematically catalogued. The results were compiled into a comprehensive database (Table 1, Table 2 and Table 3; Tables S2–S4), with each entry including historical and artistic details such as provenance, authorship (if known), current location, estimated chronology, and official institutional inventory numbers.

### 4.2. Plant Identification and Occurrence

Accurate identifications of *Verbascum* in artworks present a significant challenge due to the genus’s complex taxonomy, subtle distinguishing features, and frequent hybridization [66]. To refine our analysis, we focused on the most widely distributed species in Europe using data from the Global Biodiversity Information Facility (GBIF, https://www.gbif.org; accessed on 31 March 2025). Search parameters included: Basis of record—observation, living specimen, occurrence; Continent—Europe; Occurrence status—present; Scientific name *Verbascum* L. (https://doi.org/10.15468/4qdfhd; accessed on 31 march 2025).

For species-level identification in artworks, we applied methodologies established in previous studies [2,17,27]. Particular attention was given to diagnostic morphological features, including overall leaf structure (linear or subovate shape; simple structure, linear or undulate edges and margins, alternate arrangement, presence of basal leaves, and progressive size reduction along the stem). When present, inflorescence architecture was also considered (erect flowering stem with dense terminal racemes, spikes or panicles), as well as flower morphology (typically yellow or white corollas). To ensure accuracy, identifications were cross-referenced with botanical descriptions in Flora d’Italia [66] and Flora Europaea [67] verified using online resources with photographic documentation of live plants, such as the Dryades Project and the Royal Botanical Gardens, Kew.

For the analysis of occurrence, the single elements of the database were considered when calculating frequencies. However, when the characters of the depicted plants were uncertain or not clearly detectable, we preferred not to consider this element in the data, even if we could not securely exclude the mullein presence in the intention of the artist.

### 4.3. Interpretation of Mullein’s Symbolism Through History

To assess the symbolic significance of *Verbascum*, we first examined its potential presence and description in historical literary sources. For ancient Greek and Latin cultures, we considered references to both uses and symbolic meanings in the following key texts: *Naturalis Historia* by Pliny the Elder (23–79 AD), *De Rerum Natura* by Lucretius (98/94–50/55 BC), De Re Rustica by Varro (116–27 BC), Aeneid and Georgics by Virgil (70–19 BC), Odes by Horace (65-8 BC), Metamorphosis by Ovid (43 BC–17/18 AD), and De Materia Medica by Dioscorides (40–90 AD).

For the Renaissance and Modern periods, we considered both period-specific literary texts and scholarly works on plant symbolism in the Euro-Mediterranean art [18,37,68].

Given the strong religious focus of paintings from the Middle Ages and the Renaissance, and the central role of Christianity in shaping iconographic tradition [15], we also examined the Gospels and the Bible to explore potential symbolic associations between *Verbascum* and religious subjects in artworks.

Because symbolic associations are not always explicitly stated in these sources, we also adopted the “associative methodology” among shape and significance proposed by Caneva (2010) [27]. This approach considers that, in ancient thought, plant morphology, growth habits, and ecological preferences were often interpreted as the expression of their linkage with a specific goddess, enabling the establishment of the symbolic attribution of meaning (related to the concept of *Signatura rerum*) [37].

For each artwork included in our database, we assessed the typology of the depicted *Verbascum* elements, categorizing them into three distinct cases: Basal Leaves only (Bl), entire Vegetative system (presence of leaves and basal rosettes) (Vs), and presence of flowers in well-formed inflorescence (Inflor). This classification was designed to explore potential symbolic meanings associated with specific parts of the represented plants.

In addition, we recorded the placement of *Verbascum* in the Artwork and its main subject, also considering the symbolic relation. This allowed for a contextual analysis of the plant’s presence, enabling a more nuanced interpretation of its potential symbolic role within each artwork.

### 4.4. Statistical Analyses

After analyzing the recurrence and frequency of the different *Verbascum* species across various historical periods, we investigated possible correlations between the subject of the artwork and the specific plant parts represented. We also analyzed the relationship between artwork typology and the context of representation to identify key thematic patterns across the different historical periods considered.

All statistical analyses were performed using software R (version 4.3.1) and PAST (version 4). For graphical representation, we employed histograms to evaluate the recurrence of Verbascum species according to the considered historical periods. To illustrate the connection between the artworks, in which the plant is represented, and the Morphological elements of *Verbascum* with the Symbolic relation of the representation’s context, we used the chord diagrams applying the “circlize package” in R. In the matrix, giving the presence of some outliers in the artworks’ typologies and the symbolic relations, to reduce the possible noise, we considered them in the same category named “others”.

A map showing the provenance of all artworks and their current locations was created using QGIS (version 3.34.8).

## 5. Conclusions

By reviewing an extensive corpus of bibliographic sources and examining artworks of various types from diverse Euro-Mediterranean cultures, both in physical museums and online collection, we were able to accurately identify different species of *Verbascum* in approximately one hundred artworks. This comprehensive survey allowed us not only to assess the recurrence of this genus in art throughout history but also to interpret its symbolic significance. The study provides compelling evidence that *Verbascum*, a genus sometimes neglected in art historical literature, has held a persistent and evolving presence in European and Mediterranean visual culture, carrying a deep reservoir of symbolic and cultural meaning.

In the ancient world, *Verbascum’s* iconographic recurrence in funeral and mythological contexts, particularly in association with figures such as Demeter, Persephone, Dionysus and Hermes, reinforces its interpretation as a symbol of regeneration, protection, and the cyclical passage between life and death. Its association with Minerva/Athena, likely the origin of its symbolic value, and with Apollo further reinforces its connection to wisdom and light, linking the plant to divine knowledge and spiritual guardianship.

In the modern period, the reappearance of *Verbascum*, especially in Christian iconography, reflects both continuity and transformation of its symbolic role. The plant becomes a visual conduit for theological themes of redemption, spiritual illumination, and divine intercession, particularly in representations of Christ, St. John the Baptist, and various saints. Its frequent pairing with moments of nativity or martyrdom, corresponding to vegetative or flowering stages, respectively, highlights its role as a botanical metaphor for spiritual growth, sacrifice, and revelation.

By the eighteenth and the nineteenth centuries, while *Verbascum* continues to appear in funerary and protection contexts, its symbolism declines, occasionally functioning as a mere landscape element. This shift reflects a broader aesthetic change in the portrayal of plants, marking a transition from emblematic representation to more naturalistic observation.

Ultimately, the recurrence of *Verbascum* across diverse media, geographies, and historical periods reveals not only its enduring aesthetic presence but also its rich cultural resonance. Rooted in mythology, theology, ecological distinctiveness, and traditional medicinal knowledge, *Verbascum* emerges as a meaningful visual motif, one that invites deeper interdisciplinary analysis and challenges the notion of plants in art as purely decorative.

## Figures and Tables

**Table 1:** Artworks from the Greek and Roman Classical period featuring *Verbascum* depiction (*V. thapsus* (*) or *V. sinuatum,* reported without any symbol). Part of the plant (Elem.) depicted as Basal Leaves (Bl), Vegetative systems (Vs), Inflorescence (Inflor).

Elem.	Artwork (Context—Symbolic relation)	Date	Artwork’s Provenance—Present Location	Cit.
Bl (*)	*Akroterion* of Parthenon (Athena—light)	437–432 BC	Athens (GR)—Archaeological Museum of the Acropolis (GR)	New
Bl	Crown of a public funerary Monument (Funerary—New Life)	394/393 BC	Athens (GR)—National Archaeological Museum (GR)	New
Bl	The column of the dancers (Apollo—light)	330 BC	Delphi (GR)—Archaeological Museum (GR)	New
Bl	Corinthian Capital (Rebirth)	330 BC ca	Delphi (GR)—Archaeological Museum (GR)	New
Bl	Corinthian Capital (Rebirth)	Uncertain	Athens (GR)—National Archaeological Museum (GR)	New
Vs	Apulian painted vase (Funerary—New life)	360–350 BC	Ruvo (IT)—Jatta National Archaeological Museum (IT)	[25]
Vs	Apulian painted vase n. 1 (Funerary—New life)	340-320 BC	Ruvo (IT)—MANN, Naples (IT)	New
Vs	Apulian painted vase n. 2 (Funerary—New life)	340-320 BC	Ruvo (IT)—MANN, Naples (IT)	New
Vs	Apulian painted vase n. 1 (Funerary—New life)	340-320 BC	Canosa (IT)—MANN, Naples (IT)	New
Vs	Apulian painted vase n. 2 (Funerary—New life)	340-320 BC	Canosa (IT)—MANN, Naples (IT)	New
Vs	Apulian painted vase n. 3 (Funerary—New life)	340-320 BC	Canosa (IT)—MANN, Naples (IT)	New
Vs	Apulian painted vase n. 4 (Funerary—New life)	340-320 BC	Canosa (IT)—MANN, Naples (IT)	New
Bl	Figurative capital (Minerva—Light)	III sec. BC	Canosa (IT)—San Leucio Archaeological Park (IT)	[25]
Vs	Floral frieze (Funerary—New life)	III sec. BC	Lecce (IT)—Hypogeal Palmieri (IT)	[33]
Bl	*Athenaion* frieze (Minerva—Light)	IV sec. BC	Castro Lecce (IT)—Archaeological Museum of Castro “Antonio Lazzari” (IT)	[25]
Bl	Corinthian Capital (Rebirth)	II sec. BC	Palestrina (IT)—Sanctuary of Fortuna Primigenia (IT)	New
Bl	Corinthian Capital (Rebirth)	II sec. BC	Rome (IT)—Palatine Museum (IT)	New
Bl	Figurative capital n. 1 (Dionysus—Rebirth)	I sec. BC	Brindisi (IT)—Provincial Archaeological Museum “F. Ribrezzo” (IT)	New
Bl	Figurative capital n. 2 (Dionysus- Rebirth)	I sec. BC	Brindisi (IT)—Provincial Archaeological Museum “F. Ribrezzo” (IT)	New
Inflor (*)	Ara Pacis Frieze (Rebirth)	9 BC	Rome (IT)—Museum of the Ara Pacis (IT)	[27]
Bl	Cinerary urn (Funerary—New life)	I sec.	Rome (IT)—Centrale Montemartini Museum (IT)	New
Bl	Relief base (Dionysus—Rebirth)	I sec. BC–I sec.	Rome (IT)—National Roman Museum (IT)	New
Bl (*)	Candelabrum (Dionysus—Rebirth)	I–II sec.	Rome (IT)—Borghese Gallery (IT)	New
Bl	Frieze with “Acanthus volutes” n. 1 (Metamorphic—Never-ending life)	I sec.	Rome (IT)—Centrale Montemartini Museum (IT)	New
Bl	Frieze with “Acanthus volutes” n. 2 (Metamorphic—Never-ending life)	I sec.	Rome (IT)—Centrale Montemartini Museum (IT)	New
Vs (*)	Frieze (Metamorphic—Never-ending life)	I sec.	Rome (IT)—Vatican Museums (VA)	New
Vs	Frieze (Metamorphic—Never-ending life)	I sec.	Rome (IT)—Roman Fora (IT)	New
Bl	Statue (Isis—Rebirth)	II sec.	Rome (IT)—Borghese Gallery (IT)	New
Bl (*)	Statue of a Triple-Bodied Hecate (Hecate—Afterlife)	II sec.	Rome (IT)—Borghese Gallery (IT)	New
Vs (*)	Frieze (Metamorphic—Never-ending life)	II sec.	Rome (IT)—Roman Fora (IT)	New
Vs	Frieze (Metamorphic—Never-ending life)	II sec.	Rome (IT)—Roman Fora (IT)	New
Vs	Capital reliefs (Metamorphic—Never-ending life)	II sec.	Rome (IT)—Vatican Museums (VA)	New
Bl	Stucco relief (Solar-Light)	II sec.	Rome (IT)—Archaeological Park of Centocelle (IT)	[12]

**Table 2:** Artworks featuring *Verbascum* depictions in the Renaissance and Modern Era (*V. sinuatum* (*)*, V. nigrum* (°), *V. densiflorum* (**), while *V. thapsus* is reported without any symbol). Part of plants depicted (Elem.) are reported as Basal leaves (Bl), or inflorescence (Inflor).

Elem.	Title (Subject—Symbolic Relation)	Author	Date	Artwork’s Provenance—Present Location	Cit.
Inflor (*)	Doors of the Baptistery of St. John (St. John the Baptist)	V. Ghiberti	1453/1466	Florence (IT)—Duomo Square (IT)	[29]
Inflor (°)	Mary meets St. Elisabeth (Virgin Mary- Visitation)	Unknown	1470/1480	Wien (AUS)—Abbey Collection (AUS)	[34]
Inflor	Calvary (Christ—Crucifixion)	Antonello da Messina	1475	Messina (IT)—Royal Museum of Fine Arts Antwerp (BE)	[31]
Inflor	St. Francis in the desert (St. Francis—Ecstasy)	G. Bellini	1480	Venice (IT)—The Freak Collection (USA)	[35]
Inflor	Crocifisso Niccolini (Christ—Crucifixion)	G. Bellini	1480/1502	Venice (IT)—Palazzo degli Alberti (IT)	New
Bl	Spring (Zephyros)	S. Botticelli	1482	Florence (IT)—Uffizi Gallery (IT)	New
Bl	The Virgin of the Rocks (Christ- Child with Virgin Mary and St. John)	Leonardo da Vinci	1483/1486	Milan (IT)—Louvre Museum (FR)	New
Inflor (**)	Crucifixion of Christ (Christ—Crucifixion)	Unknown	1490/1500	Bojnice (SK)—Castle Museum (SK)	[34]
Inflor	Christ among four angels with the symbols of the Passion (Christ—Crucifixion)	V. Carpaccio	1496	Udine (IT)—Civici musei e gallerie di storia e arte (IT)	New
Bl	The Revelation of St. John (St. John the Baptist—Apocalypse)	A. Dürer	1498	Nuremberg (DE)—Staatliche Kunsthalle (DE)	New
Inflor	The Whore of Babylon, from “The Apocalypse” (Apocalypse)	A. Dürer	1498	Nuremberg (DE)—Metropolitan Museum of Art (USA)	New
Inflor	St. Giles and the Hind (St. Giles and the deer)	Master of St. Giles	1500 ca	Paris (FR)—The National Gallery (UK)	New
Bl	Visitation (Virgin Mary—Visitation)	A. Dürer	1503	Nuremberg (DE)—Metropolitan Museum of Art (USA)	New
Bl	The Madonna with the Iris (Christ-Child with Virgin Mary)	A. Dürer	1500/1510	Nuremberg (DE)—The National Gallery (UK)	New
Inflor	Death by Fire of the philosopher (Funerary—Death of philosopher)	Unknown	1500/1510	Passau (DE)—Abbey Collection (AUS)	[34]
Bl	The flight into Egypt (Christ—Child with Virgin Mary)	V. Carpaccio	1500	Venice (IT)—National Gallery of Art (USA)	New
Inflor	Pietà (Christ—Compassion)	G. Bellini	1502	Venice (IT)—Gallerie dell’Accademia (IT)	[31]
Inflor	The departure of Ceyx (Ceyx—Departure)	V. Carpaccio	1502/1507	Venice (IT)—The National Gallery (UK)	New
Bl	Great piece of turf (Still-life)	A. Dürer	1503	Nuremberg (DE)—Albertina (AT)	New
Inflor	The preparation of Christ’s tomb (Christ—Entombment)	V. Carpaccio	1505	Venice (IT)—Staatliche Museen zu Berlin (DE)	New
Inflor	The beheading of St. John the Baptist (St. John the Baptist—Death)	A. Altdorfer	1512	Nuremberg (DE)—Metropolitan Museum of Art (USA)	[36]
Inflor	The two St. Johns (St. John the Baptist)	A. Altdorfer	1512	Nuremberg (DE)—Undetermined	[36]
Inflor	The gift of the Egyptians to the Hebrews (Biblical event)	B. Luini	1514	Monza (IT)—Pinacoteca di Brera (IT)	New
Inflor	St. George and the Dragon (St. George)	L. Beck	1515	Augsburg (DE)—Kunsthistorisches Museum (AT)	New
Inflor (*)	Lion of San Marco (Lion of Venice)	V. Carpaccio	1516	Venice (IT)—Doge’s Palace (IT)	New
Inflor	The martyrdom of St. Sebastian (St. Sebastian—Martyrdom)	H. Holbein the Elder	1516	Augsburg (DE)—Alte Pinakothek (DE)	New
Inflor	Psyche Lodge (Wonder—Biodiversity)	Giovanni da Udine	1517	Rome (IT)—Villa Farnesina (IT)	[37]
Inflor	Rest on the Flight into Egypt (Christ—Child with Virgin Mary)	J. Patinir	1518/1520	Antwerp (BE)—Museo del Prado (ES)	New
Inflor	Resting in Egypt with St. Francis (Christ-Child with Virgin Mary and St. Francis)	Correggio	1520	Correggio (IT)—Uffizi Gallery (IT)	[38]
Inflor	Virgin Mary with the Child, St. John the Baptist, St. Paul and a musician angel (Christ-Child, St. John Bapt)	Uknown	1520/1530	Lombardy (IT)—Pinacoteca di Brera (IT)	New
Bl	Amor and Psyche Hall (Capital)	Giulio Romano	1524/1534	Mantova (IT)—Palazzo Te (IT)	New
Inflor	Susanna and the Elders (Susanna—Biblic Virtue)	A. Altdorfer	1526	Nurnberg (DE)—Alte Pinakothek (DE)	[39]
Inflor	Flagellation (Christ—Flagellation)	Uknown	1530/1540	Mantova (IT)—Pinacoteca di Brera (IT)	New
Inflor	St. John the Baptist in the Wilderness (St. John the Baptist)	Moretto da Brescia	1535	Brescia (IT)—Los Angeles County Museum of Art (USA)	New
Bl	Terrestrial Paradise in the Universal Cosmography (Christ—Salvation)	S. Münster	1558	Basel (CH)—Private Collection	[31]
Bl	St. Francis receives the stigmata (St. Francis—Ecstasy)	Caravaggio	1594/1595	Rome (IT)—Civici musei e gallerie di storia e arte (IT)	[31]
Bl	Rest on the flight to Egypt (Christ-Child with Virgin Mary)	Caravaggio	1597	Rome (IT)—Doria Pamphilj Gallery (IT)	[40]
Bl	St. John the Baptist (St. John the Baptist)	N. Regnier	1600 ca.	Venice (IT)—The State Hermitage Museum (RUS)	New
Bl	Deposition of Christ (Christ -Death)	Caravaggio	1600 ca.	Vatican City (SCV)—Vatican Museum (SCV)	[41]
Bl (*)	Conversion of St. Paul (St. Paul—Conversion)	Caravaggio	1600/1601	Rome (IT)—Odescalchi Balbi Collection (IT)	New
Inflor	St. John the Baptist in the desert (St. John the Baptist)	G. Vermiglio	1620	Milan (IT)—Pinacoteca Ambrosiana (IT)	[32]
Bl	St. John the Baptist (St. John the Baptist)	G. Vermiglio	1601	Rome (IT)—Pinacoteca Capitolina (IT)	[32]
Inflor	St. John the Baptist with lamb (St. John the Baptist)	G. Vermiglio	1620/1625	Milan (IT)—IIPAB (IT)	[32]
Bl	St. John the Baptist in the desert (St. John the Baptist)	G. Vermiglio	1630	Pavia (IT)—Certosa Di Pavia (PV)	New
Bl	St John the Baptist (St. John the Baptist)	Caravaggio	1602	Rome (IT)—Doria Pamphilj Gallery (IT)	[41]
Bl	St John the Baptist (St. John the Baptist)	Caravaggio	1604	Naples (IT)—Nelson-Atkins Museum of Art (USA)	[41]
Bl	The adoration of the Golden Calf (Biblic Event)	Pietro da Cortona	1612	Rome (IT)—Palazzo Mattei (IT)	New
Inflor	Noli me tangere (Christ—Resurrection)	F. Galizia	1616	Milan (IT)—Pinacoteca di Brera (IT)	New
Bl	Orpheus (Orpheus—Fascinating animals)	M. Provenzale	1618	Rome (IT)—Galleria Borghese (IT)	New
Bl	The Rape of Proserpina (Persephone)	G. L. Bernini	1621/1622	Rome (IT)—Galleria Borghese (IT)	New
Bl (*)	Apollo and Daphne (Metamorphic- New Life)	G. L. Bernini	1622/1625	Rome (IT)—Galleria Borghese (IT)	New
Bl	Saint Francis in meditation (St. Francis—Ecstasy)	C. Mellin	1624/1626	Rome (IT)—Private Collection	[42]
Inflor	The Flight into Egypt (Christ—Child and Virgin Mary)	Rembrandt	1627	Leiden (NL)—Musée des Beaux-Arts de Tours (FR)	[38]
Bl	Porcellino Fountain (Salvific)	P. Tacca	1633	Florence (IT)—New Market Square (IT)	[16]
Bl	Four Rivers Fountain (Victory of Church against Devil)	G. L. Bernini	1648/1651	Rome (IT)—Navona Square (IT)	[7]
Bl	Landscape with Sermon of St John the Baptist (St. John the Baptist)	G. F. Grimaldi	1678	Rome (IT)—Galleria Borghese (IT)	New

**Table 3:** Artworks from the eighteenth and nineteenth centuries featuring *Verbascum* (*V. thapsus* and *V. sinuatum* (+)). Part of plants depicted (Elem.) are reported as Basal leaves (Bl), or inflorescence (Inflor).

Elem.	Title (Context—Symbolic relation)	Author	Date	Artwork’s Provenance—Present Location	Citation
Bl	The tomb of Maria Flaminia Odelscalchi Chigi (Funerary)	P. Posi	1700 ca	Rome (IT)—Santa Maria del Popolo (IT)	New
Bl ^(+)^	Trevi’s Fountain (Water—Power of Nature)	N. Salvi	1732/1762	Rome (IT)—Trevi Fountain Square (IT)	[43]
Inflor	Portrait of the singer Giuditta Pasta (Landscape)	G. Molteni	1829	Milan (IT)—Pinacoteca di Brera (IT)	New
Inflor	The Goddess in the Mullein (Mythology)	J. Malczewski	1888	Krakow (PL)—Jagiellonian University Museum (PL)	Suggested by the title
Inflor	A Nymph in Mullein (Mythology)	J. Malczewski	1888	Krakow (PL)—Jagiellonian University Museum (PL)	Suggested by the title
Inflor	Mullein (Landscape)	J. Stanislawski	1887	Poland—National Museum in Krakow (PL)	Suggested by the title
Inflor	Mullein (Landscape)	J. Stanislawski	1895	Poland—National Museum in Krakow (PL)	Suggested by the title
Inflor	Portrait of a Faun (Mythology)	J. Malczewski	Unknown	Kraków (PL)—Private Collection	New

## Data Availability

Data are contained within the manuscript and Supplementary Materials.

## Supplementary Materials

The following supporting information can be downloaded at: https://www.mdpi.com/article/10.3390/plants14213294/s1, Table S1: Comparative description of the selected most widespread *Verbascum* species across Europe; Table S2: Artworks featuring *Verbascum* depictions from the ancient world (extended); Table S3: Artworks featuring *Verbascum* depictions since Renaissance to Modern Age (extended); Artworks featuring *Verbascum* depictions from the Eighteenth and Nineteenth centuries (extended); Table S4: Artworks from the Eighteenth and Nineteenth centuries with references to the species (Sp.) *V. thapsus* (T), *V. sinuatum* (S). The plants’ part depicted (Elem.) as inflorescence (Inflor), Basal leaves (Bl), the artwork typology, date, Provenance (Prov.); the position of the mullein and the Context of representation. For the previously determined presence of mullein, the references to published data (Cit.).
