# Supplementary material for: FIAT LUX: The Mullein’s (Verbascum sp.) Image and Its Symbology Through History Within the Euro-Mediterranean Culture
Source: Plants (Basel). 2025 Oct 28;14(21):3294. doi: 10.3390/plants14213294 (PMC12608489; doi:10.3390/plants14213294)
Supplement: Supplementary file 1 [file plants-14-03294-s001.zip › plants-3880488-supplementary/supplementary/Supplementary table 1.pdf]

Supplementary table 1. Comparative description of the selected most widespread *Verbascum* species across Europe, and the species more likely to be misinterpreted with *Verbascum* spp.

SPECIES	HABITUS	LEAVES	INFLORESCENCE
***Verbascum thapsus* L.**	Biennial grass of 5-12 dm; simple, densely leafy stem	Lower lanceolate leaves of 3-4x10-15 (and up to 10x30) cm, crenulate; smaller cauline leaves, at the base long decurrent on the stem and forming 2-3 cm wide wings	Inflorescences simple, cylindrical; flowers in bunches of 3-7 per bract; bracts lanceolate-acuminate 12-18 mm; flowers subsessile; corolla pale yellow to milky white
***Verbascum nigrum* L.**	Perennial grass of 6-9 dm; erect stem, striated, reddened at the top	Basal leaves with 10-15 cm petiole and triangular-lanceolate blade (8-12x15-25 cm), heart-shaped at the base; upper cauline leaves lanceolate (1-2x3-6 cm) subsessile	inflorescence generally simple, white-woolly bracts and calyxes; flowers in bunches of 3-7 per bract; linearbracts 3-7(-10) mm; peduncles 4-8(-12) mm; corolla diameter 18-20 mm
***Verbascum lychnitis* L.**	Biennial grass of 8-15 dm; stem branched at the top and thick also at the base	Basal leaves with lanceolate blade (5-8x15-25 cm), long narrowed into a 1-5 cm petiole, irregularly toothed on the edge	Branched inflorescence with flowers in bunches of 3-7 at the axil of each bract; lower bracts lanceolate and upper linear; peduncles 6-11 mm; yellow or whitish corolla, diameter (12-)15(-20) mm.
***Verbascum sinuatum* L.**	Biennial grass of 4-10 dm; trunks with abundant arching branches	Basal leaves 4-10x12-22cm, sessile, lobed or divided with 4-5 incisions per side and coarsely toothed; cauline leaves ovate-acuminate or more or less lanceolate	Inflorescence widely branched; flowers in bunches of 3-7 per bract; bracts (3.8 mm) with heart-shaped base and elongated and thin apex; peduncles 2-4 mm; corolla diameter 15-30 mm
***Verbascum densiflorum* Bertol.**	Biennial herb of 5-10 dm; simple stem	Basal leaves lanceolate-linear, very sharp, regularly serrated; cauline leaves decurrent for 1-2 cm along the stem.	Inflorescence simple or slightly branched; flowers in bunches of 3-7 per bract; triangular-acuminate bracts (15-40 mm); subnull peduncles

***Verbascum pulverulentum* Vill.**	Biennial grass of 5-12 dm; trunks with arched branches	Basal leaves generally serrated or crenulate, sessile, about twice as long as they are wide, the upper ones broadly heart-shaped, with entire margin, short tip.	Flowers in bunches of 3-7 per bract; linear bracts (3-5 mm); corolla diameter 18-25 mm
***Verbascum blattaria* L.**	Biennial grass of 4-7(-12) dm	Basal leaves spatulate, subsessile or with a 2-3 cm petiole and a lanceolate blade of 1-3x4-6(-10) cm, leaf margin always more or less toothed, in the basal leaves the teeth are generally deep and are added to an irregular lobulation (up to 1/3 of the blade); cauline leaves sessile, linear-lanceolate (1-2x4-8 cm), serrated, semi-embracing, non-decurrent.	Branchy inflorescence, with single flowers at the axil of each bract; yellow corolla, violet at the base, diameter 20-30mm; peduncles 6-10mm
***Verbascum phlomoides* L.**	Biennial grass of 3-10 dm; simple stem	Basal leaves lanceolate-spatulate 4-11x15-30 cm, obtusely and irregularly toothed, subsessile or with a winged petiole of 2-10 cm; upper leaves sessile and sumi-amplexicaul, not decurrent or extending on about 1/5 of the distance with the previous leaf	Dense inflorescence, simple or slightly branched; flowers in bunches of 3-7 per bract; ovate-acuminate bracts of 5-6x10-14 mm; flower peduncles 4-10 mm, at fruiting 5-15 mm; corolla diameter 40-50 mm
***Acanthus mollis* L.**	Perennial grass of 5-12 dm; cylindrical, erect, simple, subwoody stems	leathery basal leaves with spatulate perimeter (1.5-2x5-8 dm), pinnately divided with 6-7 deep incisions on each lobe, petiolate, not spiny	Cylindrical spike (3x30 cm); 3-bracts: the central one is ovate with 7-9 tips, 5-nerve, the lateral ones are linear-flacate, single-nerve, ending in a sharp point; pinkish-white corolla with only the trilobed lower lip of 35x45 mm, shorter than the upper calyceal lip
***Plantago major* L.**	Perennial grass	Leaves all in basal rosette, close to the ground, (5-)7(-9)-nerves, dark green, with a broadly oval-shaped lamina, 1.1-3 times longer than wide; entire margin, base almost truncated (right angle with the petiole) or cordate; petiole 0.3-1.2 times longer than the blade, grooved above and winged, especially below	scape briefly curved at the base then erect, spike tapering towards the apex
