# Supplementary material for: FIAT LUX: The Mullein’s (Verbascum sp.) Image and Its Symbology Through History Within the Euro-Mediterranean Culture
Source: Plants (Basel). 2025 Oct 28;14(21):3294. doi: 10.3390/plants14213294 (PMC12608489; doi:10.3390/plants14213294)
Supplement: Supplementary file 1 [file plants-14-03294-s001.zip › plants-3880488-supplementary/supplementary/Supplementary table 2.pdf]

Supplementary table 2: List of artworks featuring *Verbascum* depictions from the ancient world, with references to the species (Sp.) *V. thapsus* (T), *V. sinuatum* (S), Not determined (ND). The plants' part depicted (Elem.) as Leaf, Vegetative systems (Vs), inflorescence (Inflor), Basal leaves (Bl), the artwork typology, date, Provenance, the position of the mullein and the representation context. For the previously determined presence of mullein, the published data references (Cit.).

Sp.	Elem.	Artwork	Position	Context	Date	Artwork's Provenance	Cit.
T	Bl	*Akroterion* of Parthenon	Central-like supermodel	Athena	437-432 BC	Athens (GR)	New
S	Bl	Crown of a public funerary Monument	Central-like supermodel	Funerary	394/393 BC	Athens (GR)	New
S	Bl	The column of the dancers	Base of the column	Apollo	330 BC	Delphi (GR)	New
S	Bl	Corinthian Capital	Base of the capital	Rebirth	330 BC ca	Delphi (GR)	New
S	Bl	Corinthian Capital	Base of the capital	Rebirth	Uncertain	Athens (GR)	New
S	Vs	Apulian painted vase	Base of winged figure	Funerary [24]	360–350 BC	Ruvo (IT)	[25]
S	Vs	Apulian painted vase n.1	Base of central head and handles	Funerary [24]	340-320 BC	Ruvo (IT)	New
S	Vs	Apulian painted vase n.2	Base of central head and handles	Funerary [24]	340-320 BC	Ruvo (IT)	New
S	Vs	Apulian painted vase n.1	Base of central head and handles	Funerary [24]	340-320 BC	Canosa (IT)	New
S	Vs	Apulian painted vase n.2	Base of winged figure and handles	Funerary [24]	340-320 BC	Canosa (IT)	New
S	Vs	Apulian painted vase n.3	Base of central head and handles	Funerary [24]	340-320 BC	Canosa (IT)	New
S	Vs	Apulian painted vase n.4	Base of central head and handles	Funerary [24]	340-320 BC	Canosa (IT)	New
S	Bl	Figurative capital	Base of female central head	Minerva	III sec. BC	Canosa (IT)	[25]
ND	Vs	Floral frieze	ND	Funerary	III sec. BC	Lecce (IT)	[33]
ND	Bl	*Athenaion* frieze	ND	Minerva	IV sec. BC	Castro Lecce (IT)	[25]
S	Bl	Corinthian Capital	Base of capital	Rebirth	II sec. BC	Palestrina (IT)	New
S	Bl	Corinthian Capital	Base of capital	Rebirth	II sec. BC	Rome (IT)	New
S	Bl	Figurative capital n. 1	Next to deities' heads	Dionysus [Guadalupi 2019]	I sec. BC	Brindisi (IT)	New
S	Bl	Figurative capital n.2	Next to deities' heads	Dionysus [Guadalupi 2019]	I sec. BC	Brindisi (IT)	New
Cfr. T	Inflor	Ara Pacis Frieze	Candelabrum-like supermodel	Metamorphic	9 BC	Rome (IT)	[27]
S	Bl	Cinerary urn	Lid of cinerary urn	Funerary	I sec.	Rome (IT)	New

S	Bl	Relief base	Next to winged dancers	Dionysus	I sec. BC -I sec.	Rome (IT)	New
T	Bl	Candelabrum	Base of candelabrum	Dionysus	I-II sec.	Rome (IT)	New
S	Bl	Frieze with “*Acanthus* volutes” n.1	Base of winged sphynx	Metamorphic	I sec.	Rome (IT)	New
S	Bl	Frieze with “*Acanthus* volutes” n.2	Part of metamorphic element	Metamorphic	I sec.	Rome (IT)	New
T	Vs	Frieze	In the center, generating stems of Vitis vinifera	Metamorphic	I sec.	Rome (IT)	New
S	Vs	Frieze	At the base of the generative central element	Metamorphic	I sec.	Rome (IT)	New
S	Bl	Statue	On the head of Isis	Isis	II sec.	Rome (IT)	New
T	Bl	Statue of a Triple-Bodied Hecate	Part of *kalathos*	Hecate	II sec.	Rome (IT)	New
T	Vs	Frieze	Part of candelabrum-like figure	Metamorphic	II sec.	Rome (IT)	New
S	Vs	Frieze	Part of candelabrum-like element	Metamorphic	II sec.	Rome (IT)	New
S	Vs	Capital reliefs	Base of capital, generating a stem inflorescence	Metamorphic	II sec.	Rome (IT)	New
S	Bl	Stucco relief	ND	Solar light [12]	II sec.	Rome (IT)	[12]

12. Kumbaric, A.; Bartoli, F.; Hosseini, Z.; Bellini, A.; Caneva, G. Botanical Representations from Villa Della Piscina Di Centocelle (Rome, Italy) for a Reconstruction of Ancient Roman Gardens. *Acta IMEKO* **2024**, *13*, 1–8, doi:10.21014/actaimeko.v13i4.1857.

24. Heuer, K. Tenacious Tendrils: Replicating Nature in South Italian Vase Painting. In Proceedings of the Arts; MDPI, 2019; Vol. 8, p. 71.

25. Caneva, G.; Lazzara, A.; Albani Rocchetti, G.; D’Andria, F. Plant Biodiversity and Its Meaning at the Origin of the Peopled Scrolls Friezes: Data from the Castrum Minervae Sanctuary (Castro- Lecce, Italy). *Rend. Fis. Acc. Lincei* **2024**, *35*, 1077–1093, doi:10.1007/s12210-024-01286-1.

27. Caneva, G. *Il codice botanico di Augusto. The Augustus botanical code*; Gangemi Editore: Rome, 2010; ISBN 978-88-492-1933-3.

33. L’Arab, G. L’ipogeo Palmieri di Lecce. *mefr* **1991**, *103*, 457–497, doi:10.3406/mefr.1991.1725.
