# Supplementary material for: FIAT LUX: The Mullein’s (Verbascum sp.) Image and Its Symbology Through History Within the Euro-Mediterranean Culture
Source: Plants (Basel). 2025 Oct 28;14(21):3294. doi: 10.3390/plants14213294 (PMC12608489; doi:10.3390/plants14213294)
Supplement: Supplementary file 1 [file plants-14-03294-s001.zip › plants-3880488-supplementary/supplementary/Supplementary table 3.pdf]

Supplementary table 3. List of artworks featuring *Verbascum* depictions since Renaissance to Modern Age with references to the species (Sp.) *V. thapsus* (T), *V. sinuatum* (S), V. *densiflorum* (D), *V. nigrum* (N), Not determined (ND). The plants' part depicted (Elem.) as Leaf, Vegetative systems (Vs), inflorescence (Inflor), Basal leaves (Bl), Infruttescence (Frut) the artwork typology, date, Provenance (Prov.); the position of the mullein and the representation Context. For the previous determinated presence of mullein, the references of published data (Cit.).

Sp.	Elem.	Artworks	Title	Author	Position	Context	Date	Artwork's Provenance – Present Location	Cit.
S	Inflor	Gate of baptistery	Doors of the Baptistery of St. John	V. Ghiberti	Architrave (Frame)	St. John the Baptist	1453/1466	Florence (IT)	[29]
N	Inflor	Painting	Mary meets St. Elisabeth	Unknown	At the back of St. Elisabeth	Virgin Mary (Visitation)	1470/1480	Wien (AUS)	[34]
T	Inflor	Painting	Calvary	Antonello da Messina	Under the cross	Christ (Crucifixion)	1475	Messina (IT)	[31]
T	Inflor	Painting	St. Francis in the desert	G. Bellini	At the back of St. Francis	St. Francis (Ecstasy)	1480	Venice (IT)	[35]
T	Inflor	Painting	Crocifisso Niccolini	G. Bellini	Under the cross	Christ (Crucifixion)	1480/1502	Venice (IT)	New
T	Bl	Painting	Spring	S. Botticelli	Under Mercury	Zephyros	1482	Florence (IT)	New
T	Bl	Painting	The Virgin of the Rocks	Leonardo da Vinci	Near Christ child	Christ (Child with Virgin Mary and St. John)	1483/1486	Milan (IT)	New
D	Inflor	Painting	Crucifixion of Christ	Unknown	At the back of the cross	Christ (Crucifixion)	1490/1500	Bojnice (SK)	[34]
T	Inflor	Painting	Christ among four angels with the symbols of the Passion	V. Carpaccio	Beneath the angels	Christ (Crucifixion)	1496	Udine (IT)	New
T	Bl	Xilography	The revelation of St. John: 9. St. John devours the book	A. Dürer	At the back of St. John	St. John the Baptist (Apocalypse)	1498	Nurnberg (DE)	New
T	Inflor	Woodcut	The Whore of Babylon, from "The Apocalypse"	A. Dürer	At the feet of the whore of Babylon	Apocalypse	1498	Nurnberg (DE)	New
T	Inflor	Painting	St. Giles and the Hind	Master of St. Giles	In front of St. Giles holding the deer	St. Giles (and the deer)	1500 ca	Paris (FR)	New
T	Bl	Woodcut	Visitation	A. Dürer	Under the two women	Virgin Mary (Visitation)	1503	Nurnberg (DE)	New
T	Bl	Painting	The Madonna with the Iris	A. Dürer	At the back of Virgin Mary holding the child	Christ (Child with Virgin Mary)	1500/1510	Nurnberg (DE)	New
T	Inflor	Painting	Death by Fire of the philosopher	Unknown	Front of image	Funerary (Death of philosopher)	1500/1510	Passau (AUS)	[34]
T	Bl	Painting	The flight into Egypt	V. Carpaccio	Under Virgin Mary holding the baby	Christ (Child with Virgin Mary)	1500	Venice (IT)	New
T	Inflor	Painting	Martinengo Pietà	G. Bellini	At the back of the Virgin Mary holding Christ (Pietà)	Christ (Compassion)	1502	Venice (IT)	[31]

T	Inflor	Painting	The departure of Ceyx	V. Carpaccio	Near the couple	Ceyx (Departure)	1502/1507	Venice (IT)	New
T	Bl	Watercolor	Great piece of turf	A. Dürer	Front of image	Still-life	1503	Nurnberg (DE)	New
T	Inflor	Painting	The preparation of Christ's tomb	V. Carpaccio	Front of the scene	Christ (Entombment)	1505	Venice (IT)	New
T	Inflor	Painting	The beheading of St. John the Baptist	A. Altdorfer	Near the execution of St. John the Baptist	St. John the Baptist (Death)	1512	Nurnberg (DE)	[36]
T	Inflor	Woodcut	The two St. Johns	A. Altdorfer	Near St. John	St. John the Baptist	1512	Nurnberg (DE)	[36]
T	Inflor	Fresco	The gift of the Egyptians to the Hebrews	B. Luini	Front of image	Biblical event	1514	Monza (IT)	New
T	Inflor	Painting	St. George and the Dragon	L. Beck	Near the female figure	St. George (and the dragon)	1515	Augsburg (DE)	New
S	Inflor	Painting	Lion of San Marco	V. Carpaccio	Near the lion	Lion of Venice	1516	Venice (IT)	New
T	Inflor	Painting	The martyrdom of St. Sebastian	H. Holbein the Elder	At the feet of St. Sebastian	St. Sebastian (Martyrdom)	1516	Augsburg (DE)	New
Cfr. T	Inflor	Fresco	Psyche Lodge	Giovanni da Udine	Part of vegetal festoon	Wonder (Biodiversity)	1517	Rome (IT)	[37]
T	Inflor	Painting	Rest on the Flight into Egypt	J. Patinir	Near Virgin Mary with child	Christ (Child with Virgin Mary)	1518/1520	Antwerp (BE)	New
Cfr. T	Inflor	Painting	Resting in Egypt with St. Francis	Correggio	At the back of St. Francis	Christ (Child with Virgin Mary and St. Francis)	1520	Correggio (IT)	[38]
T	Inflor	Painting	Virgin Mary with the Child, St. John the Baptist, St. Paul and a musician angel	Uknown	Under St. John the Baptist	Christ (Child, St. John Bapt)	1520/1530	Lombardy (IT)	New
T	Bl	Architectonic element	Amor and Psyche Hall	Giulio Romano	Base of capital	(Capital)	1524/1534	Mantova (IT)	New
T	Inflor	Painting	Susanna and the Elders	A. Altdorfer	Near Susanna holding lilies	Susanna (Biblic Virtue)	1526	Nurnberg (DE)	[39]
T	Inflor	Painting	Flagellation	Uknown	Behind Christ	Christ (Flagellation)	1530/1540	Mantova (IT)	New
T	Inflor	Painting	St. John the Baptist in the Wilderness	Moretto da Brescia	Behind St. John the Baptist	St. John the Baptist	1535	Brescia (IT)	New
T	Bl	Xilography	Terrestrial Paradise in the Universal Cosmography	S. Münster	ND	Christ (Salvation)	1558	Basel (CH)	[31]
T	Bl	Painting	St. Francis receives the stigmata	Caravaggio	Under St. Francis in estasy	St. Francis (Ecstasy)	1594/1595	Rome (IT)	[31]
T	Bl	Painting	Rest on the flight to Egypt	Caravaggio	Under Virgin Mary holding the baby	Christ (Child with Virgin Mary)	1597	Rome (IT)	[40]
T	Bl	Painting	St. John the Baptist	N. Regnier	Next to St. John the Baptist	St. John the Baptist	1600 ca.	Venice (IT)	New
T	Bl	Painting	Deposition of Christ	Caravaggio	Under Christ	Christ Death	1600 ca.	Vatican City (SCV)	[41]

S	Bl	Painting	Conversion of St. Paul	Caravaggio	Near St. Paul	St. Paul Conversion	1600/1601	Rome (IT)	New
T	Inflor	Painting	St. John the Baptist in the desert	G. Vermiglio	Under St. John the Baptist	St. John the Baptist	1620	Milan (IT)	[32]
T	Bl	Painting	St. John the Baptist	G. Vermiglio	Under St. John the Baptist	St. John the Baptist	1601	Rome (IT)	[32]
T	Inflor	Painting	St. John the Baptist with lamb	G. Vermiglio	Under St. John the Baptist	St. John the Baptist	1620/1625	Milan (IT)	[32]
T	Bl	Painting	St. John the Baptist in the desert	G. Vermiglio	Under St. John the Baptist	St. John the Baptist	1630	Pavia (IT)	New
T	Bl	Painting	St John the Baptist	Caravaggio	Under St. John the Baptist	St. John the Baptist	1602	Rome (IT)	[41]
T	Bl	Painting	St John the Baptist	Caravaggio	Under St. John the Baptist	St. John the Baptist	1604	Naples (IT)	[41]
ND	Bl	Painting	The adoration of the Golden Calf	Pietro da Cortona	ND	Biblic Event	1612	Rome (IT)	New
T	Inflor	Painting	Noli me tangere	Fede Galizia	Next to Maddalena in Noli me tangere	Christ (Resurrection)	1616	Milan (IT)	New
T	Bl	Mosaic	Orpheus	M. Provenzale	Under Orpheus	Orpheus (Fascinating animals)	1618	Rome (IT)	New
T	Bl	Marble sculpture	The Rape of Proserpina	G. L. Bernini	Under Cerberus	Persephone	1621/1622	Rome (IT)	New
S	Bl	Marble sculpture	Apollo and Daphne	G. L. Bernini	Under Apollo and Daphne	Metamorphic (New Life)	1622/1625	Rome (IT)	New
T	Bl	Painting	Saint Francis in meditation	C. Mellin	Under St. Francis	St. Francis (Ecstasy)	1624/1626	Rome (IT)	[42]
T	Inflor	Painting	The Flight into Egypt	Rembrandt	ND	Christ (Child and Virgin Mary)	1627	Leiden (NL)	[38]
S	Bl	Bronze sculpture	Porcellino Fountain	Pietro Tacca	Under the boar	Salvific	1633	Florence (IT)	[16]
T	Bl	Fountain	Four Rivers Fountain	G. L. Bernini	Near the snake	Victory of Church against Devil	1648/1651	Rome (IT)	[7]
T	Bl	Painting	Landscape with Sermon of St John the Baptist	G. F. Grimaldi	Under St. John the Baptist	St. John the Baptist	1678	Rome (IT)	New

7. Caneva, G.; Altieri, A.; Kumbaric, A.; Bartoli, F. Plant Iconography and Its Message: Realism and Symbolic Message in the Bernini Fountain of the Four Rivers in Rome. *Rend. Fis. Acc. Lincei* **2020**, *31*, 1011–1026, doi:10.1007/s12210-020-00946-2.

16. Clauser, M.; Grigioni, A.; Nepi, C. Identification of the Plants in Artworks: The Bronze Base of the "Fountain of the Porcellino" of Florence. *Atti della Società Toscana di Scienze Naturali Residente in Pisa Memorie serie B* **2012**, 65–67, doi:10.2424/ASTSN.M.2012.09.

29. Di Vito, M. La Cornice Bronzea Della Porta Sud Del Battistero Di San Giovanni a Firenze: Una Proposta Di Lettura. Master Thesis, Università degli Studi di Firenze, Scuola di specializzazione in Storia dell'Arte. Indirizzo Arte Minori: Unpublished, 2006.

31. Papa, R. Tasso barbasso: soluzione dell'enigma Available online: https://www.formaelucis.com/museo-diocesano/tasso-barbasso-soluzione-dellenigma (accessed on 13 June 2025).
32. Pavesi, M. Un Nuovo "San Giovanni Battista Nel Deserto" Della Fase Caravaggesca Di Giuseppe Vermiglio. *Arte Lombarda* **2010**, 14–19.
34. Sillasoo, Ü. Landscapes, Vegetation, and Folklore in Late Medieval Art: An Iconographic Study Based on Selected Austrian and South German Panel Paintings. *Landscape Research* **2014**, *39*, 455–479, doi:10.1080/01426397.2012.761188.
35. Lavin, M.A.; Liu, J.; Gitner, A. The Joy of St. Francis: Bellini's Panel in the Frick Collection. *Artibus et Historiae* **2007**, *28*, 231–256.
36. White, E.M. Albrecht Altdorfer's Botanical Attribute for Saint John the Baptist. *Source: Notes in the History of Art* **1996**, *15*, 15–21, doi:10.1086/sou.15.2.23205516.
37. Caneva, G. *Meraviglia, amore e potere: le pitture botaniche più ricche del mondo nella Loggia di Psiche di Raffaello e Giovanni da Udine: Villa La Farnesina, Roma = Wonder, love and power: the botanical paintings more comprehensive in the world in the Lodge of Psyche by Raphael and Giovanni da Udine, Villa La Farnesina, Rome*; Nardini editore: Firenze, 2022; ISBN 978-88-404-0622-0.
38. Conti, S. Simboli nell'arte, il Tasso Barbasso – Restauro e gestione cantieri Available online: http://www.silviaconti.it/simboli-nellarte-il-tasso-barbasso/ (accessed on 13 June 2025).
39. Bischoff, C. Albrecht Altdorfer's Susanna and the Elders: Female Virtues, Male Politics. *racar* **2020**, *23*, 22–35, doi:10.7202/1073291ar.
40. Brown, B.L. Travellers on the Rocky Road to Paradise: Jacopo Bassano's Flight into Egypt. *Artibus et Historiae* **2011**, 193-219,279-280.
41. McTighe, S. Caravaggio's Physiognomy. In *Representing from Life in Seventeenth-century Italy*; Amsterdam University Press, 2020 ISBN 978-90-485-3326-8.
42. Primarosa, Y. Nuove Proposte per Charles Mellin Pittore e disegnatore lorenese a Roma. *Bollettino d'Arte* 53–76.
