# Supplementary material for: FIAT LUX: The Mullein’s (Verbascum sp.) Image and Its Symbology Through History Within the Euro-Mediterranean Culture
Source: Plants (Basel). 2025 Oct 28;14(21):3294. doi: 10.3390/plants14213294 (PMC12608489; doi:10.3390/plants14213294)
Supplement: Supplementary file 1 [file plants-14-03294-s001.zip › plants-3880488-supplementary/supplementary/Supplementary table 4.pdf]

Supplementary table 4. Artworks from the Eighteenth and Nineteenth centuries with references to the species (Sp.) *V. thapsus* (T), *V. sinuatum* (S). The plants' part depicted (Elem.) as inflorescence (Inflor), Basal leaves (Bl), the artwork typology, date, Provenance (Prov.); the position of the mullein and the Context of representation. For the previously determined presence of mullein, the references to published data (Cit.).

Sp.	Elem.	Artwork	Title	Author	Position	Context	Date	Prov.	Citation
S	Bl	Funerary monument	The tomb of Maria Flaminia Odelscalchi Chigi	Paolo Posi	At the base of the tree	Funerary	1700 ca	Santa Maria del Popolo (IT)	New
T+S	Bl	Fountain	Trevi's Fountain	N. Salvi	In the lower part	Water (Power of Nature)	1732/1762	Trevi Fountain Square (IT)	[43]
S	Inflor	Painting	Portrait of the singer Giuditta Pasta in the stage costume of 'Nina o sia la pazza per amore'	G. Molteni	At the side of Giuditta Pasta	Landscape	1829	Pinacoteca di Brera (IT)	New
S	Inflor	Painting	The Goddess in the Mullein	J. Malczewski	Central figure	Mythology	1888	Jagiellonian University Museum (PL)	Suggested by the title
S	Inflor	Painting	A Nymph in Mullein	J. Malczewski	Central figure	Mythology	1888	Jagiellonian University Museum (PL)	Suggested by the title
S	Inflor	Painting	Mullein	J. Stanislawski	In lateral close-up	Landscape	1887	National Museum in Krakow (PL)	Suggested by the title
Cfr. T	Inflor	Painting	Mullein	J. Stanislawski	In lateral close-up	Landscape	1895 ca	National Museum in Krakow (PL)	Suggested by the title
S	Inflor	Painting	Portrait of a Faun	J. Malczewski	Retrospective of the faun	Mythology	Unknown	Private Collection	New

43. Caneva, G.; Dinelli, A. Analisi Della Iconografia Botanica e Della Flora Ruderale per Lo Studio e La Conservazione Della Fontana Di Trevi. In *Fontana di Trevi: la storia, il restauro*; Cardilli, L., Quilici, L., Ioppolo, G., Eds.; Carte segrete: Roma, 1991; pp. 191–200 ISBN 978-88-85203-43-3.
